# Metabolic Reprogramming in COVID-19

**DOI:** 10.3390/ijms222111475

**Published:** 2021-10-25

**Authors:** Tao Shen, Tingting Wang

**Affiliations:** 1 The State Key Laboratory of Pharmaceutical Biotechnology, Division of Immunology, Medical School, Nanjing University, Nanjing 210093, China; 181230029@smail.nju.edu.cn; 2 Jiangsu Key Laboratory of Molecular Medicine, Division of Immunology, Medical School, Nanjing University, Nanjing 210093, China

**Keywords:** COVID-19, metabolic changes, tryptophan, arginine, glucose, cholesterol, fatty acids

## Abstract

Plenty of research has revealed virus induced alternations in metabolic pathways, which is known as metabolic reprogramming. Studies focusing on COVID-19 have uncovered significant changes in metabolism, resulting in the perspective that COVID-19 is a metabolic disease. Reprogramming of amino acid, glucose, cholesterol and fatty acid is distinctive characteristic of COVID-19 infection. These metabolic changes in COVID-19 have a critical role not only in producing energy and virus constituent elements, but also in regulating immune response, offering new insights into COVID-19 pathophysiology. Remarkably, metabolic reprogramming provides great opportunities for developing novel biomarkers and therapeutic agents for COVID-19 infection. Such novel agents are expected to be effective adjuvant therapies. In this review, we integrate present studies about major metabolic reprogramming in COVID-19, as well as the possibility of targeting reprogrammed metabolism to combat virus infection.

## 1. Introduction

Viruses are morbific pathogens which rely on host cell machinery to achieve their entry, replication, maturation and dissemination [1]. Therefore, it is understandable that viruses have evolved to induce metabolic reprogramming in host cells to create a hospitable environment for their survival [2]. Different virus species tend to trigger different metabolic alternations in order to satisfy their unique requirement for successful spread [3]. While the complex mechanism of host–virus interaction remains to be revealed, it is evident that metabolic reprogramming is closely associated with outcomes of viral infections, emphasizing their potential role in developing antiviral agents [4].

The COVID-19 pandemic, whose causative agent is severe acute respiratory syndrome coronavirus 2 (SARS-CoV-2), places an extraordinary burden on public health [5]. Acute respiratory distress syndrome (ARDS), a characteristic of many critical COVID-19 cases, is likely to arise from immune dysfunction which is featured with cytokine storm [6]. Metabolic diseases such as diabetes and obesity have long been established to cause immune dysfunction, with impairment to both the innate and adaptive immune system [7]. As a result, it is not difficult to understand the high prevalence of diabetes, obesity, and risk factors for cardiovascular disease in critical COVID-19 cases [8]. Given the confirmed association between metabolic diseases and the severity of COVID-19 infection, as well as the close relation between immune response and metabolism alternations, metabolic reprogramming in COVID-19 pathophysiology has attracted considerable interest [8,9]. In this review, we gather the present understanding about metabolic reprogramming in COVID-19 and discuss how the exploitation of a reprogrammed metabolism offers novel therapeutic paradigms.

## 2. Metabolism Reprogramming in COVID-19 Infection

### 2.1. Amino Acid Metabolism

As amino acid metabolism plays a significant part in the regulation of immune responses and the assembly of progeny virus [10,11,12], alternations of amino acid metabolism over the course of SARS-CoV-2 infection has become one of the focuses in COVID-19 research. In this article, we will review the changes in the tryptophan, arginine and glutamine metabolisms of COVID-19 patients, which have been subject to more extensive research.

#### 2.1.1. Tryptophan Pathways

Tryptophan (Trp), an essential amino acid, has four known downriver metabolism pathways: the kynurenine, decarboxylation and transamination and serotonin pathways [13]. An overwhelming percentage of tryptophan is catabolized along the kynurenine pathway and generates a variety of metabolites with recognized biological roles in regulating immune responses [14,15]. In the kynurenine pathway, the generation of N-formylkynurenine derived from Trp is the rate determining step via the enzyme activity of indoleamine-2,3-dioxygenase 1 (IDO1), IDO2 and tryptophan-2,3-dioxygenase (TDO) [16]. Consumption of Trp weakens the immune reactions by enhancing regulatory T cells (Treg) activity, and inhibiting T effector cells [17,18].

In COVID-19 infection, kynurenine-to-tryptophan ratio is increased, suggesting the activation of the kynurenine pathway [19]. The increase of kynurenine and several downstream products, as well as the decrease in tryptophan, serotonin, and indolepyruvate levels, which validates the alternation of the kynurenine pathway, has also been revealed by several metabolomic analyses [20,21,22,23,24,25]. Notably, the changes in the kynurenine pathway correlate with disease severity [19,21,25]. This can be explained as follows. Activation of the kynurenine pathway may be a result of excessive inflammatory responses in COVID-19 patients, given interferon (IFN)-γ and other inflammatory factors can upregulate IDO [26,27]. The immunosuppressive effects arising from the hyperactivation of the kynurenine pathway might further delay the clearance of SARS-CoV-2 and cause cytokine storm and multiorgan failure. In accordance with general kynurenine pathway changes mentioned before, IDO-Kyn-AhR pathway is activated by IFN-β or IFN-γ in alveolar epithelial cells, leading to an accumulation of mucins, thus triggering hypoxia of COVID-19 [28].

Therefore, the hyperactivation of the kynurenine pathway provides a potent explanation of the COVID-19 pathological process, indicating a potential therapeutic approach by targeting the tryptophan pathway. Among the pharmacological agents targeting tryptophan pathways, IDO inhibitors are the most clinically advanced, which prevent immune suppression caused by tryptophan depletion and kynurenine metabolites [29,30]. However, no clinical trials have been conducted concerning IDO inhibitors in COVID-19 infection. Further research is expected to explore their effects.

#### 2.1.2. Arginine Reprogramming

Arginine, a semi-essential amino acid, is primarily involved in both the generation of proteins and the production of metabolites by the catalysis of four sets of enzymes: arginine:glycine amidinotransferase, arginases, NO synthases, and arginine decarboxylase [31]. In the case of metabolic or traumatic stress, arginine is depleted, despite the fact that it can be endogenously synthesized [32]. Arginine starvation suppresses T cell activity by blocking cyclin D3 and cyclin-dependent kinase, which are fundamental in regulating T-cell-cycle progression [33]. In contrast, elevating arginine levels not only improve metabolic fitness of activated T cells, but also promote T cell survival through the action of three transcriptional regulators [34].

Consistent with the pattern mentioned above, COVID-19 patients present with acute arginine depletion, which is significantly associated with T cell defects [25,35]. Arginine supplements restore T cells’ ability of proliferation ex vivo among COVID-19 patients [25]. These findings suggest arginine supplementation may be an adjuvant therapy, which is discussed below.

A number of researchers have shown that arginine supplements are beneficial to a diversity of critically ill populations with clinical safety [36]. Two clinical trials concerning arginine supplements in COVID-19 are underway (NCT04404426, NCT04637906). Interestingly, as a key metabolite for viral replication, arginine depletion has also been proposed as a potential therapeutic approach for treating COVID-19 patients [37]. The balance between antiviral and immune suppression effects should be taken into consideration to confirm the optimal dosing window and proper timing of intervention for arginine-related treatment in the course of COVID-19.

#### 2.1.3. Glutamine Metabolism

Glutamine, a non-essential amino acid, has diverse physiological roles, such as energy generation, lipid synthesis, purine synthesis and glutathione production [38]. Moreover, glutamine has also been revealed as a necessary nutrient for the functions of immune cells, including lymphocyte, macrophage and neutrophil, all of which are important for immune protection against viral infection [39]. Given the consumption of glutamine by human organs and immune cells increases considerably when subjected to stressful conditions, glutamine can also be condition-essential [40,41].

As glutamine metabolism provides metabolic intermediates that are required for virus assembly, many viruses are likely to increase glutamine metabolism to support their replication [12]. A similar situation occurs in SARS-CoV-2 infection; COVID-19 patients have a significantly reduced glutamine to glutamate ratio, indicating increased utilization of glutamine [22]. Considering the aforementioned role of glutamine in purine synthesis and increased folate and one carbon metabolism for the synthesis of purine in SARS-CoV-2 infected cells, enhanced glutamine metabolism in COVID-19 infection may be a result of rising demand for purine synthesis, which is critical for progeny virus assembly [42]. However, inconsistent with other virus infections where glutamine replenishes the TCA cycle for the generation of energy and lipids, incorporation of glutamine into oxidized TCA cycle is reduced in SARS-CoV-2 infection, decreased expression of α-ketoglutarate dehydrogenase complex may be a possible explanation [38,43].

Synthesized from glutamate, cysteine, and glycine, glutathione (GSH) plays an important role in antioxidant defense and cell proliferation [44]. Detection of SARS-CoV-2 infected cells 8 h after infection showed increased GSH accompanied by a depletion in folate, raising the possibility that the antioxidant role of glutathione facilitates SARS-CoV-2 replication, protecting SARS-CoV-2 infected cells from reactive oxygen species (ROS) induced death, thus enabling the initial virus replication [42]. However, blocking glutathione synthesis did not change the progress of SARS-CoV-2 infection, suggesting increased GSH may be an epiphenomenon or can only function in a microenvironment in vivo [42]. In contrast, the detection of SARS-CoV-2 infected cells 24 h after infection indicated impaired biosynthesis of GSH [45]. A possible explanation is that increased methylation demands caused by SARS-CoV-2 infection give priority to utilize substrates for glutathione synthesis to generate methyl-groups [46]. Of note, decreased GSH may lead to the massive accumulation of ROS, which induce cell death and assist the release and spread of viral particles [47]. More research is needed to disclose dynamic changes in GSH and the specific functions of GSH in SARS-CoV-2 replication.

**Figure 1:**
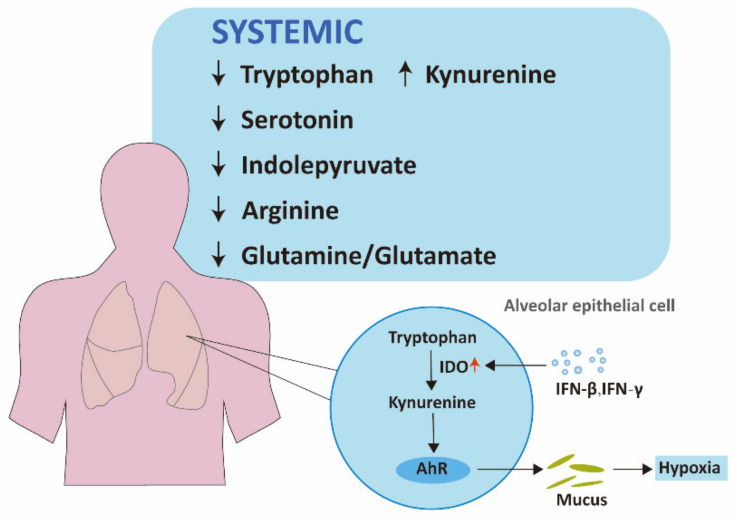
Altered tryptophan and arginine metabolism in COVID-19 patients. In COVID-19 infection, tryptophan metabolism is upregulated through kynurenine pathway. Mucus production is stimulated via IDO-Kyn-AhR pathway, resulting in hypoxia of COVID-19. Arginine depletion and increased glutamine metabolism is also discovered in COVID-19 patients. IFN, interferon; IDO, indoleamine-2,3-dioxygenase; AhR, aryl hydrocarbon receptor.

To summarize, glutamine metabolism plays a vital part in SARS-CoV-2 infection and is a potential therapeutic target in COVID-19 infection. Regarding the fact that glutamine is essential for the functions of immune cells, glutamine supplements have been proposed as an adjunctive therapy. A study including 60 COVID-19 patients, with half of the patients using L-Glutamine, showed L-Glutamine supplements in the early stage of SARS-CoV-2 infection improved prognosis of COVID-19 patients [48]. Nevertheless, inhibitors of glutamine metabolism are also put forward to be beneficial in SARS-CoV-2 infection as they can deplete substrates for purine synthesis and accordingly suppress SARS-CoV-2 replication. Glutamine inhibitors that target only the SARS-CoV-2 infected cells are expected to be developed to assist in the fight of COVID-19 infection (Figure 1).

### 2.2. Glucose Metabolism and HIF

Under normoxic, most differentiated cells metabolize glucose into pyruvate, thus feeding the mitochondrial tricarboxylic acid (TCA) cycle. Nicotinamide adenine dinucleotide (NADH) is then produced through the TCA cycle, fueling oxidative phosphorylation (OXPHOS) for the maximum generation of ATP. It is only under hypoxia that pyruvate is directly converted into lactate[49]. Comprised of HIF-1 α and HIF-1 β subunits, hypoxia-inducible factor 1 (HIF-1) plays a huge part in the shift of metabolism toward anaerobic glycolysis [50]. In contrast, lactate fermentation is the chief driver of energy production in cancer cells, regardless of oxygen level. Such metabolism change is called aerobic glycolysis, or the Warburg effect [49,51].

Many viruses are reported to enhance glycolysis, which allows the rapid production of energy and other substrates necessary for viral replication [3]. Consistent with such mode, metabolomic, proteomic and transcriptomic analyses have revealed increased levels of pyruvate, pyruvate kinase and lactate dehydrogenase (LDH) in COVID-19, all of which are metabolites or enzymes of glycolysis, indicating enhanced glucose metabolism for lactate fermentation [52,53,54,55]. Glycolysis is of vital importance for SARS-CoV-2 to replicate [56,57]. Increased glycolysis in monocyte not only sustains cytokine production including TNF-α, IL-1β, and IL-6, but also brings about T cell impairment and lung epithelial cell death [57].

HIF-1 α is a powerful inductor of glycolysis in COVID-19. Enzymes involved in glycolysis, including hexokinase 1/hexokinase 3, aldolase A, pyruvate kinase 2, and lactate dehydrogenase A/lactate dehydrogenase B, were significantly upregulated by HIF-1 signaling [54]. Several studies have identified the upregulation of HIF 1α [55,57]. Both hypoxia and reactive oxygen species (ROS) mediate HIF-1 α stabilization [57,58]. However, comprehensive mechanism accounting for increased HIF-1α in COVID-19 infection remains unclear. The research focusing on AKT/mTOR/HIF-1 signaling shows reduced expression of HIF-1α, which is inconsistent with the studies described before [55,57]. Notwithstanding activation of Akt-mTOR pathway, HIF-1 α expression is suppressed in SARS-CoV-2 infected cell line Huh7 [59]. Further research is indispensable to disclose the role of Akt-mTOR signaling in the regulation of HIF-1 α expression in SARS-CoV-2 infection.

Besides, HIF-1 α plays a significant regulatory role in immune response[60]. As a transcriptional suppressor of interferon regulatory factor (IRF)5, HIF-1 α suppresses type I; interferon, partly explaining the impaired IFN production in severe COVID-19 infections [61].

Given the close connection between increased glycolysis and inflammation response in COVID-19 infection, inhibiting glycolysis is emerging as an attractive therapeutic modality. Here, we discuss the potential therapeutic effects of a ketogenic diet (KD) and HIF inhibitors.

KD, a high fat but low carbohydrate diet, is proposed to switch the metabolism from glycolysis to ketogenesis by lowering the availability of glucose [62]. Through activation of protective effect of γδ T cell responses, KD restrains infection of coronavirus and influenza virus in mice [63,64]. Retrospective analysis has shown clinical efficacy of KD in COVID-19 cytokine storm [65]. Randomized controlled trial (NCT04492228) is in progress to confirm the preliminary data.

**Figure 2:**
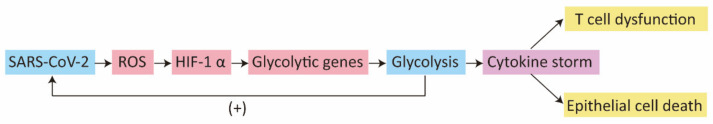
Schematic view of how SARS-CoV-2 enhances glycolysis and subsequent pathological change. SARS-CoV-2 induces glycolysis in a ROS/HIF-1α dependent way. Increased glycolysis fosters replication of SARS-CoV-2 and cytokine storm, giving rise to T cell impairment and lung epithelial cell death. ROS, reactive oxygen species; HIF, hypoxia-inducible factor.

Concerning the crucial part of HIF-1 α in enhanced glycolysis and immune regulatory activities in SARS-CoV-2 infection, treatment efficacy of HIF inhibitors is worthy of examination. Plenty of clinical trials have inquired into the therapeutic implication of HIF inhibitors on cancers, providing convenience for research on HIF-1 inhibitors in COVID-19 [66] (Figure 2).

### 2.3. Cholesterol Metabolism

Cholesterol is an essential lipid for appropriate cellular and systemic functions. Mainly localized to cell membranes, cholesterol not only regulates the rigidity and fluidity of the membrane, but also modulates the conformations of transmembrane proteins [67]. The homeostasis of cholesterol metabolism depends on the dynamic equilibrium between uptake, de novo biosynthesis, conversion, esterification and export [68]. Cholesterol is obtained either from exogenous uptake from the diet or endogenous synthesis [69]. In addition to its aforementioned role in cell membranes, cholesterol provides a substrate for the production of bile acids, steroid hormones and vitamin D. Oxysterols, which are formed in the first step of the conversion, have aroused great interest due to its multiple biological role [70]. Redundant cellular cholesterol is esterified to cholesteryl esters by cholesterol acyltransferase (ACAT), most of which are thus stored in cytoplasmic lipid droplets [71]. Exported to the blood, cholesteryl, esters can also serve as a chief component of plasma lipoproteins, including very-low-density lipoproteins (VLDLs), low-density lipoproteins (LDLs) and high-density lipoproteins (HDLs) [67].

Cholesterol is important for the entry and replication of SARS-CoV-2. Spike protein of SARS-CoV-2 interacts with HDL or its components, facilitating SARS-CoV-2 entry in a way dependent on ACE2 through scavenger receptor B type 1 (SR-B1) [72]. Membrane cholesterol is essential for the fusion of SARS-CoV-2 spike protein in a raft-independent way [73]. Accumulation of lipid droplets (LDs), which reserve cholesterol esters and triacylglycerols to fulfil diverse intentions, is a distinct characteristic of SARS-CoV-2 infected cells [74,75]. Inhibition of LD formation significantly suppresses SARS-CoV-2 replication, demonstrating the essential role of cholesterol metabolic reprogramming in SARS-CoV-2 pathogenesis [74].

**Figure 3:**
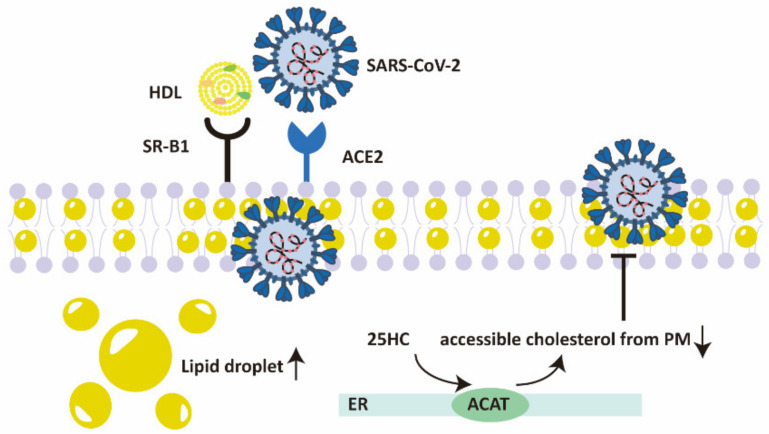
The role of cholesterol in regulating the entry of SARS-CoV-2. HDL-scavenger receptor B type 1 (SR-B1) facilitates SARS-CoV-2 entry in an ACE2-dependent manner. Accumulation of lipid droplets is a marked characteristic of SARS-CoV-2 infected cells. 25HC depletes accessible cholesterol from plasma membrane by stimulating the ER-localized ACAT, thus suppressing membrane fusion of SARS-CoV-2. HDL, high-density lipoprotein; SR-B1, scavenger receptor B type 1; ACE2, angiotensin-converting enzyme 2; 25HC, 25-hydroxycholesterol; ER, endoplasmic reticulum; ACAT, acyl-CoA:cholesterol acyltransferase.

Cholesterol 25-hydroxylase (CH25H) is an interferon-stimulated gene (ISG) which encodes the enzyme that produces oxysterol 25-hydroxycholesterol (25HC) from cholesterol [76]. The level of CH25H is increased in two SARS-CoV-2 infected lung epithelial cell lines ex vivo, as well as macrophages and epithelial cells in COVID-19 patients [77]. Depleting membrane cholesterol by stimulating the ER-localized acyl-CoA:cholesterol acyltransferase (ACAT), 25HC suppresses membrane fusion and thus inhibits SARS-CoV-2 replication [77]. In line with these findings, another research confirms the antiviral function of 25HC in SARS-CoV-2 and provides the explanation of the mechanism as blockade of cholesterol export [78]. Besides, oxysterol 27-hydroxycholesterol (27HC) is also reported to pose an antiviral activity against SARS-CoV-2 [79] (Figure 3).

A decrease in cholesterol level has been revealed by a quantity of researchers since the outbreak of COVID-19. The main sterol precursor of cholesterol, namely desmosterol and lathosterol, are remarkably decreased in moderate and severe COVID-19 patients [79]. Several retrospective reviews of COVID-19 patients have found a significant decrease in total cholesterol (TC), LDL-C and HDL-C [80,81,82,83], all of which are correlated with disease severity and are predictors of clinical prognosis [84,85,86,87]. Muti-omic analyses as well as clinical research has also uncovered the downregulation of multiple lipoproteins, especially apolipoproteinA-1 (APOA1) in COVID-19 patients [20,22,87,88,89]. Remarkably, only 27HC has been noted to decrease in COVID-19 patients among the three oxysterols, i.e., 24-hydroxycholesterol (24HC), 25HC and 27HC [79]. In symptomatic COVID-19 patients, 25HC is even slightly increased [79]. The elevation of 25HC in a fatal COVID-19 case and hACE2 mice infected with SARS-CoV-2 is indicated in another study [90]. As CH25H is an ISGs, IFN mediated production of 25HC may be a possible explanation for the elevation of 25HC.

Various mechanisms are proposed to give an explanation for the decrease in cholesterol in COVID-19 patients. A basic understanding is SARS-CoV-2 consumption of cholesterol, given the vital function of cholesterol in SARS-CoV-2 entry and replication. Evidence confirming the understanding is the aforementioned fact that the decrease in cholesterol is connected with disease severity, which is usually linked to a higher virus load [91]. Another explanation is HDL-mediated depletion of cholesterol is an inflammatory state, which causes accretion of the acute phase protein serum amyloid A (SAA) within HDL and leads to increased clearance of HDL [92]. Based on these findings, HDL is speculated to consume cholesterol during COVID-19 infection [81]. Deeper investigations are imperative for the verification of the speculation. Liver damage, inflammatory cytokines, free radicals as well as alternations of vascular permeability may contribute to hypolipidemia in COVID-19 [82]. Despite these hypotheses, there is a growing interest in reprogramming cholesterol metabolism in SARS-CoV-2 infected cells.

Ordinarily, the majority of viruses tend to increase lipid synthesis to obtain enough substrates and energy for virus replication [2,4], whereas RNA sequencing analysis of human airways and alveolar organoids infected with SARS-CoV-2 showed down-regulation of several proteins related to cholesterol metabolism, including fatty acid-binding proteins (FABPs) 1/2/6, APOA1/4, APOB, APOC4 [93]. It is well known that sterol regulatory element binding protein-2 (SREBP-2) is activated under decreased cholesterol levels in serum to maintain cholesterol homeostasis [94]. SREBP2 is highly activated in COVID-19 patents’ peripheral blood mononuclear cells (PBMCs) [95]. However, up-regulation of cholesterol synthesis-suppressing genes Sestrin-1 and PCSK9 suppress SREBP-2-induced cholesterol biosynthesis in COVID-19 patents’ PBMCs [95]. More questions remain to be solved as to how cellular cholesterol metabolism changes during SARS-CoV-2 infection and in what way the decreased cholesterol level affects SARS-CoV-2 infection.

Using a genome-scale CRISPR loss-of–function screen and single-cell transcriptomics, collective transcriptional alternations in the synthesis of cholesterol next to deprivation of six genes are identified inhuman alveolar epithelial cells infected with SARS-CoV-2 [96]. CRISPR-driven deprivation of the six genes (RAB7A, PIK3C3, NPC1, CCDC22, ATP6V1A, and ATP6AP1) results in the induction of cholesterol synthesis [96]. Amlodipine, a calcium-channel antagonist that upregulates intercellular cholesterol level, leads to reduced SARS-CoV-2 viral infection [96]. Induction of the cholesterol biosynthesis pathway is implied as a prospective means to restrain SARS-CoV-2 infection in another research [97].

Taken together, the adjustment of cholesterol metabolism is an appealing therapeutic method in COVID-19 infection. Statins, inhibitors of the important cholesterol synthetic enzyme HMG-CoA reductase, are a category of lipid-lowering drugs with widespread availability, low cost and great safety [98]. Besides their lipid-lowering activity, stains are known for antithrombotic, anti-inflammatory and immunomodulatory properties [99,100]. As a result, statin therapy is expected to be a supplementary therapy for COVID-19 [101]. A series of studies analyzed the effect of statin use on prognosis in COVID-19 infection. A large retrospective analysis on 13,981 COVID-19 patients in Hubei, China, indicated in-hospital statin use significantly decreases all-cause mortality [102]. Statin-associated improved prognosis is partially attributed to the relieved inflammatory response [102]. A number of other researchers have confirmed the beneficial role of statin-use in COVID-19 infection [103,104,105,106,107,108,109,110,111]. Of note, an updated meta-analysis of 22 studies suggested stains use in COVID-19 patients were connected to an approximately 35% decrease in the adjusted risk of mortality [112]. In spite of these encouraging outcomes of statin use in COVID-19, conflicting results were reported. A meta-analysis of 9 studies which includes 3449 COVID-19 patients in total revealed no improvement of clinical outcomes after statin use [113]. Another nationwide cohort study in Denmark suggested uniformity in all-cause mortality between statin users and nonusers in COVID-19 patients [114]. In view of the lack of randomize controlled trail, statin is currently recommended only if there is an indication [115]. From the point of cholesterol reprogramming in COVID-19 infection, particular attention should be given to the potentially beneficial role of statin in depleting cholesterol needed for the entry and replication of SARS-CoV-2, as well as the prospective disadvantage of leading to low level cholesterol, which is associated with a worse clinical prognosis (Table 1).

### 2.4. Fatty Acid Metabolism

Similar to cholesterol, synthesis of fatty acids is also of critical significance for the synthesis of membranes and replication of viruses [116,117]. Two key metabolic enzymes in fatty acid biosynthesis are Acetyl-CoA carboxylase (ACC) and fatty acid synthase (FASN). ACC converts acetyl-CoA to malonyl CoA, which is then transferred to fatty acid synthesis (FAS) [118]. Using acetyl-CoA and malonyl CoA as substrates, FASN catalyzes the elongation of fatty acids [119]. Expression of ACC and FASN are regulated by SREBP-1 [120]. Fatty acids are converted back into acetyl-CoA through fatty acid oxidation (FAO), which is also known as β-oxidation [121].

Activation of PI3K/AKT/mTOR/S6K signaling activity in SARS-CoV-2 infection is likely to increase ACC and FASN, thus facilitating fatty acid biosynthesis to provide enough substrates for replication [59,122]. Upregulation of SREBP1 after SARS-CoV-2 infection indicates enhanced fatty acid metabolism [74]. Transcriptome data from SARS-CoV-2 infected Calu-3 human lung epithelial cells showed hyperactivation of biological functions associated with fatty acids synthesis [123]. Increase in fatty acids abundance in COVID-19 patients have been found in a number of research; hydrolysis of phospholipids catalysed by phospholipase A2 (PLA2) is proposed as a potential explanation for increased fatty acids [124,125]. Remarkably, arachidonic acid and oleic acid were reliable predictors of disease severity [124]. Inhibitors of up- and downstream of FASN as well as FASN knockout resulted in suppression of SARS-CoV-2 replication, supply of fatty acid after FASN knockout rescued damaged SARS-CoV-2 replication [126]. Proteomic analysis of autopsy samples from COVID-19 patients indicated activation of FAO in most organs, implying reprogramming to a more efficient energy production mode [127]. Taken together, these findings suggest the essential part of fatty acids metabolism in SARS-CoV-2 replication, offering an appealing therapeutic target.

**Figure 4:**
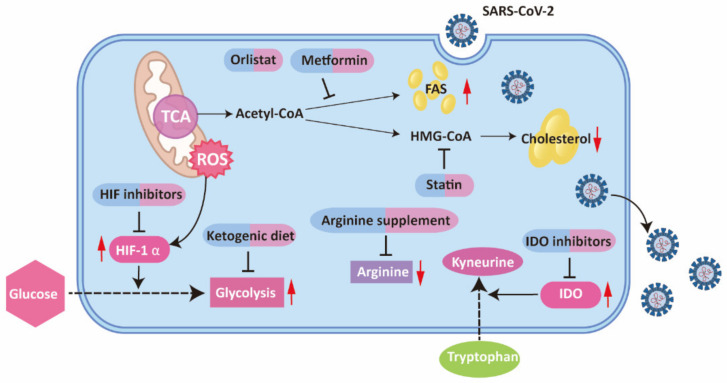
Metabolic reprogramming and corresponding therapeutic approaches in COVID-19. In COVID-19, tryptophan metabolism, glycolysis and fatty acid metabolism are upregulated, while cholesterol and arginine are decreased. Therapeutic agents targeting the reprogrammed metabolism in COVID-19 are shown in the figure. TCA, trichloroacetic acid; ROS, reactive oxygen species; HIF, hypoxia-inducible factor; FAS, fatty acid synthesis; HMG-CoV, 3-hydroxy-3-methylglutaryl-coenzyme A; IDO, indoleamine-2,3-dioxygenase.

As common medicines with well-established safety, orlistat and metformin are proposed as potential anti-SARS-CoV-2 treatments on the basis of their respective role in inhibiting FASN and activating AMPK, which cause the decrease of fatty acid synthesis for impaired replication complex formation [122]. Randomized controlled clinical trials are required to verity their efficacy (Figure 4).

## 3. Conclusions and Future Direction

Metabolic reprogramming plays multiple roles in COVID-19 infection, not only offering energy and essential substrates for SARS-CoV-2 replication, but also regulating immune response. From this perspective, COVID-19 can be understood as a metabolic disease, and both pathology and therapeutics can center on reprogrammed metabolism. Such novel treatments are currently being examined and are anticipated to be powerful adjuvant therapies for COVID-19 infection.

It is worth pointing out that pre-existing metabolism derailments may fire up metabolic reprograming in COVID-19 infection due to different levels of available nutrition and influence on immune response. A typical example of the effects of different levels of nutrition is the increased mortality in COVID-19 patients with diabetes or hyperglycemia. Increased COVID-19 mortality caused by diabetes or hyperglycemia might be a result of elevated glucose levels, which provides massive substrates for enhanced glycolysis, which subsequently generates energy and components for SARS-CoV-2 replication [57,128]. As for the influence on immune response, patients with metabolic diseases such as diabetes and obesity often respond to infection with a proinflammatory patterns rather than a protective pattern, which may lead to increased production of cytokines and ROS in COVID-19 infection, fueling the IFN-IDO-Kyn pathway and ROS-Glycolysis axis [129]. As a result, future research targeting metabolic alternations in COVID-19 infections should pay special attention to pre-existing metabolism derailments in order to achieve a deeper understanding of metabolic reprograming in the pathophysiology of COVID-19.

## Figures and Tables

**Table 1:** Retrospective studies on COVID-19-associated hypolipidemia.

Study Population	Lipid Changes in COVID-19	References
519 severe COVID-19 patients from the West Court of Union Hospital in Wuhan, China	Decrease of TC and HDL-C in non-survivors vs survivors	[80]
114 COVID-19 patients from Wenzhou Central Hospital, in Wenzhou, China	Decrease of TC, LDL-C and HDL-C in COVID-19 patients vs healthy controls Decrease of HDL-C in severe vs common groups	[81]
597 COVID-19 patients from the Cancer Center, Union Hospital of Tongji Medical College, Wuhan	Decrease of TC, LDL-C and HDL-C in COVID-19 patients vs healthy controls Decrease of TC and LDL-C in with disease severity	[82]
115 COVID-19 patients from Union Hospital of Tongji Medical College affiliated Huazhong University of Science and Technology	Decrease of HDL-C in COVID-19 patients vs healthy controls	[83]
228 COVID-19 patients from Public Health Treatment Center of Changsha, China	Decrease of TC, LDL-C and HDL-C in COVID-19 patients vs healthy controls Decrease of HDL-C in with disease severity	[84]
17 surviving and 4 non-surviving COVID-19 patients from Zhongnan Hospital of Wuhan University in Wuhan, China	Decrease of TC, LDL-C and HDL-C in non-survivors vs survivors Decrease of LDL-C in with disease severity	[85]
99 COVID-19 patients from Leishenshan Hospital in Wuhan, China.	Decrease of HDL-C in with disease severity	[87]

TC, total cholesterol; LDL-C, low-density lipoprotein cholesterol; HDL-C, high-density lipoprotein cholesterol.
